# Acceptance of Open-Label Placebos Is Linked to Holistic Health Beliefs and Thinking Styles

**DOI:** 10.3390/bs16020198

**Published:** 2026-01-29

**Authors:** Arved Seibel, Albert Wabnegger, Anne Schienle

**Affiliations:** Clinical Psychology, University of Graz, 8010 Graz, Austria

**Keywords:** open-label placebo, acceptance, health beliefs, thinking styles, holistic

## Abstract

Open-label placebos (OLPs) have demonstrated benefits across multiple conditions; however, the factors influencing their acceptance or rejection remain unclear. This study aimed to examine the relationship between willingness to take an OLP pill and variables such as thinking styles and health beliefs. We conducted an online survey in Austria with 326 participants (mean age = 33.5 years; 77% female; 57% university students). Given the divergent attitudes toward OLPs, we performed a binary logistic regression analysis (*n* = 241), excluding participants with intermediate acceptance scores to more clearly distinguish between acceptance and rejection. The results indicated that holistic thinking styles and holistic health beliefs were associated with the willingness to take an OLP. Due to the correlational nature of the study, causal inference is not possible.

## 1. Introduction

Open-label placebos (OLPs) are placebos administered without deception, whereby recipients are explicitly informed that the treatment they receive (e.g., a pill) contains no active pharmacological ingredient. The treatment procedure is typically accompanied by a rationale explaining how placebos can produce beneficial effects through various mechanisms, such as learning experiences, positive expectations, and mind–body interactions (Colloca & Howick, 2018). This OLP approach has demonstrated promising effects across a range of conditions (von Wernsdorff et al., 2021; Buergler et al., 2023).

However, acceptance of OLPs varies substantially. While some studies suggest that OLPs are generally well received (e.g., Bernstein et al., 2019; Kaptchuk et al., 2010; Krockow et al., 2023; Sandler & Bodfish, 2008), others report skepticism or even outright rejection. For example, in an online survey, approximately one third of participants reported being unwilling to take an OLP pill themselves or to administer one to their child (Schienle & Seibel, 2024). Similarly, in a study employing a case vignette of a patient with insomnia, 30% of participants indicated that they would decline the proposed OLP treatment (Haas et al., 2021). Moreover, several surveys suggest that laypeople perceive OLP treatment as counterintuitive (Haas et al., 2021; Locher et al., 2021) and often believe that deception is necessary for a placebo to be effective (Hull et al., 2013).

Given these divergent attitudes, it is important to better understand the factors that influence acceptance of OLPs. Several state- and trait-level factors have already been identified. Individuals are more likely to accept OLPs when they expect or hope that the treatment will be effective (Druart et al., 2023; Haas et al., 2021, 2022; Schienle & Seibel, 2024). In addition, curiosity and open-mindedness appear to be positively associated with OLP acceptance (Haas et al., 2022). In contrast, individuals with higher levels of health anxiety or chronic illness tend to be less willing to engage in OLP treatment (Krockow et al., 2023; Haas et al., 2021). Finally, OLP acceptance is more likely when individuals perceive the treatment as low risk and report higher levels of trust in their healthcare provider (Druart et al., 2023).

These findings suggest that specific cognitive–attitudinal constructs reflecting individuals’ beliefs about health and healthcare may be central factors influencing OLP acceptance. Accordingly, the present study focused on participants’ beliefs in holistic health and complementary/alternative medicine (CAM) versus evidence-based biomedical interventions, as well as on their tendencies toward medical maximization versus minimization.

Holistic health and CAM approaches emphasize the connection between mind and body, which is consistent with placebo mechanisms (Barrett et al., 2003). Individuals who endorse holistic health beliefs may view placebos as a natural means of harnessing the body’s self-healing capacities. Supporting this, research indicates that people who perceive the mind and body as interconnected are more likely to understand how placebos can elicit positive effects (Bishop et al., 2014). Furthermore, it has been proposed that alternative medicine may serve as a particularly effective placebo-generating healthcare approach, as it leverages nonspecific but beneficial effects of the patient–practitioner relationship, including attention and modifications of patients’ expectations (Kaptchuk, 2002).

Individuals also differ in their approach to healthcare, ranging along a continuum from medical minimization to medical maximization. Medical maximizers tend to take action, seeking healthcare proactively and often pursuing extensive interventions even when not strictly necessary. In contrast, medical minimizers adopt a more passive approach, engaging with healthcare services only when absolutely required (Mott et al., 2021; Scherer et al., 2020; Thorpe et al., 2024). Given their proactive engagement and openness to treatments, medical maximizers may be more receptive to OLPs.

In addition to health- and healthcare-related attitudes, the present study also examined more general thinking styles, such as intuitive versus analytical thinking (Pennycook, 2017). Research indicates that individuals rely on these styles to varying degrees and that preferences for one style over the other tend to be stable over time (Stagnaro et al., 2018). Compared with intuitive thinkers, analytical thinkers are less prone to superstitious, conspiratorial, and religious beliefs (Pennycook et al., 2015). They are also more skeptical of pseudoscientific claims or those that lack empirical evidence (Aßmann & Betsch, 2023; Evans et al., 2020). Consequently, intuitive thinkers might be more open to OLP treatments.

Finally, another placebo-relevant construct is dispositional optimism—the general tendency to hold positive expectations about the future (Carver & Scheier, 2014). Higher levels of optimism have been linked to increased placebo responsiveness (e.g., Kern et al., 2020; but also see Kang et al., 2023). Accordingly, individuals with greater optimism may also be more likely to accept OLP treatment.

The abovementioned research has examined only a limited number of correlates and predictors of OLP acceptance. However, preliminary evidence suggests that healthcare attitudes (Druart et al., 2023) as well as factors related to thinking styles, such as open-mindedness (Haas et al., 2022), are relevant in this context. The present study therefore aimed to extend these investigations and addressed this gap in the literature.

## 2. Materials and Methods

### 2.1. Participants

We conducted a completely anonymized online survey, advertised through social media, research platforms, and mass emails to students at a local university. Prior to participation, all individuals were informed about the structure and objectives of the study, and they provided informed consent. The average completion time was approximately 15 min. Data collection took place between June and the end of October 2024.

Inclusion criteria were an age of 18 years or older and fluency in the German language. The survey was accessed a total of 534 times; 340 participants completed it. Of these, eight were excluded because they did not consent to data publication or completed the survey in less than five minutes, and six were excluded because they did not indicate biological sex (sex was considered a predictor in the binary regression analysis). As a result, the final sample comprised 326 participants (mean age [M] = 33.5 years; standard deviation [SD] = 13.2; 77% female; 57% university students).

For the binary regression analysis, we used a subset of participants with divergent responses concerning OLP acceptance (see Section 2.3). This sample included 241 participants (M = 33.7 years; SD = 14.2; 76% female; 56% university students).

### 2.2. Measures

#### 2.2.1. Open-Label Placebo Acceptance

After receiving the definition of an OLP, participants rated their willingness to take OLP pills for physical ailments (first item) and psychological conditions (second item). Responses were recorded using slider scales ranging from 0 (refusal) to 100 (willingness).

#### 2.2.2. The Holistic Complementary and Alternative Medicine Questionnaire (HCAMQ)

The HCAMQ (Hyland et al., 2003) consists of two subscales. The holistic health subscale (five items) measures beliefs about the relationship between the body and the mind and the extent to which psychological factors affect health (e.g., “Positive thinking can help you fight off a minor illness”). The CAM subscale (six items) assesses belief in and support for CAM (e.g., “Complementary medicine builds up the body’s own defenses, leading to a permanent cure”). Responses were given on six-point scales ranging from “strongly disagree” to “strongly agree.” Higher scores indicate higher holistic health beliefs or greater CAM support.

#### 2.2.3. The Medical Maximizer–Minimizer Elicitation Question (MM1)

The MM1 (Scherer & Zikmund-Fisher, 2020) assesses preferences for active vs. passive healthcare using a single item (“Do you tend to act in unclear situations, or do you prefer to wait and see if action is necessary?”). Responses were given on a six-point scale reaching from “I strongly lean toward waiting and seeing” to “I strongly lean toward taking action.”

#### 2.2.4. The Optimism–Pessimism Short Scale-2 (SOP2)

The SOP2 (Nießen et al., 2022) measures dispositional optimism and pessimism with two items. A composite optimism score was calculated by averaging the optimism item score and the reversed-scored pessimism item.

#### 2.2.5. The 4-Component Thinking Style Questionnaire (4-CTSQ)

The 4-CTSQ (Newton et al., 2024) assesses the degree to which an individual engages in four distinct thinking styles: preference for intuitive thinking (PIT, e.g., “When I make predictions, I tend to rely on my intuition”), preference for effortful thinking (PET, e.g., “Reasoning things out carefully is not one of my strong points”; inverted), close-minded thinking (CMT, e.g., “Truth is never relative”), and actively open-minded thinking—the tendency to change one’s mind when encountering evidence that challenges existing beliefs (AOT, e.g., “Whether something is true is more important than evidence”; inverted). Each subscale consists of six statements rated on a six-point scale ranging from “strongly disagree” to “strongly agree.” For each subscale, an average score was calculated. Higher average scores indicate a stronger preference for the respective thinking style.

Detailed scale descriptions with item wording and scoring information are provided in the Supplementary Materials.

### 2.3. Statistical Analysis

We computed an average OLP acceptance score (willingness to take an OLP for physical and psychological ailments) because of the substantial correlation between the two variables (Rho = 0.76, Spearman–Brown coefficient = 0.87) in the total sample (*n* = 326), indicating a general tendency toward OLP acceptance.

**Figure 1:**
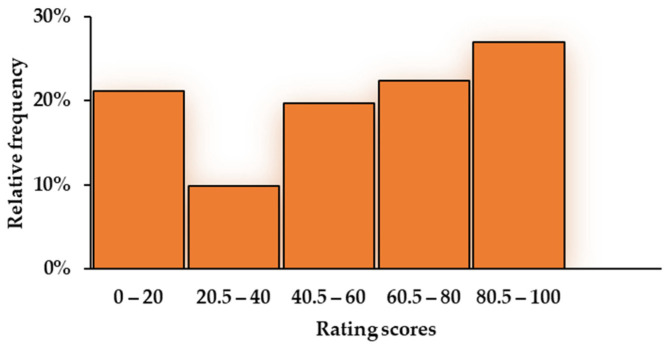
Histogram of open-label placebo acceptance scores.

The mean OLP acceptance score was 55.46 (SD = 32.98). Approximately 46% of participants had scores of ≥66, indicating OLP acceptance, whereas 28% had scores of ≤33, indicating OLP rejection. The distribution of acceptance scores was U-shaped (skewness: −0.40; kurtosis: −1.08), with a mode of 0 (see Figure 1). Therefore, Spearman’s correlation coefficients were computed to assess the relationship between the OLP acceptance score and the other measures (e.g., thinking styles, attitudes toward alternative medicine and holistic health, optimism, and medical maximizing–minimizing).

To conduct a binary regression analysis for the criterion OLP acceptance vs. rejection, we recoded OLP acceptance scores ≤ 33 as 0 (rejection) and scores ≥ 66 as 1 (acceptance), yielding a total of 241 datasets for the analysis. The selected cut-off values were chosen to ensure a clear distinction between acceptance and rejection while retaining as many cases as possible from the total sample. Variables were entered hierarchically. The first block included control variables such as sex and age, while the second block included the questionnaire measures. All metric predictors were mean-centered (each value subtracted by the sample mean) to make the intercept more interpretable.

All analyses were conducted using Jamovi Version 2.6.17 and IBM SPSS Statistics Version 29.0. Listwise deletion was used for all analyses. A significance level of α = 0.05 was applied.

## 3. Results

Descriptive statistics and Spearman’s correlation coefficients are provided in Table 1. The findings revealed that OLP acceptance was significantly correlated with all assessed variables. A robustness check including the six participants, who did not report their biological sex showed that the bivariate correlations remained stable.

Assumption checks for binary logistic regression indicated no extreme outliers, no severe deviations from the linearity of predictors with the log odds, and no multicollinearity among predictors. Variance inflation factors (VIFs) ranged from 1.03 to 1.60. In the first step, the model was significant (χ^2^[2] = 8.42; *p* = 0.015) and explained 3% of the variance in willingness to take an OLP (Table 2). The model showed rather weak discrimination capabilities (AUC = 0.64) and poor calibration as indicated by the Hosmer–Lemeshow test (χ^2^[8] = 22.31, *p* = 0.004). Adding the remaining predictors in the second step increased the explained variance to 18% (χ^2^[10] = 57.20; *p* < 0.001; ΔR^2^ = 0.15, Δχ^2^[8] = 48.78, *p* < 0.001). This second model showed good discrimination (AUC = 0.77) with no evidence of poor calibration according to Hosmer–Lemeshow test (χ^2^[8] = 5.42, *p* = 0.712).

Average marginal effects indicated that OLP acceptance was positively associated with holistic health beliefs (AME = 0.03, CI [0.01, 0.04], *p* = 0.004) and negatively associated with closed-minded thinking (AME = −0.09, CI [−0.15, −0.02], *p* = 0.007), effortful thinking (AME = −0.09, CI [−0.16, −0.02], *p* = 0.016), and actively open-minded thinking (AME = −0.08, CI [−0.14, −0.01], *p* = 0.025). Although preference for intuitive thinking showed a positive bivariate correlation with OLP acceptance, its coefficient in the multiple regression was negative but not significant, likely due to shared variance with the other thinking style predictors.

## 4. Discussion

This study examined the acceptance of OLP treatment along with associated attitudinal and cognitive factors. Consistent with previous studies, divergent attitudes toward OLPs were observed, with a significant proportion of respondents either endorsing or rejecting their use (Haas et al., 2021; Schienle & Seibel, 2024).

The willingness to take an OLP was associated with a more holistic health perspective and a less dominant analytical thinking style. Holistic health approaches emphasize the interconnectedness of the body, mind, and spirit—a concept fundamental to the mechanisms underlying placebo effects. Specifically, placebos are thought to enhance the connection between the mind and body, shaping their interaction (Kaptchuk, 2024). Similar conceptualizations of placebos are prevalent in the general population and are often linked to the notion of “the power of the mind” (Bishop et al., 2014; Locher et al., 2021; Tandjung et al., 2014).

The idea that mere belief can have tangible and profound effects on the physical world also plays a significant role in religion and spirituality. Previous research has shown that individuals who engage in spiritual practices or hold strong spiritual beliefs often experience enhanced placebo effects (Hyland et al., 2006; Kohls et al., 2011; Schienle et al., 2021). For instance, a study found that religious participants who drank tap water labeled as originating from the sanctuary in Lourdes—a major Catholic pilgrimage site known for reports of miracle cures—reported pleasant bodily sensations such as warmth and tingling (Schienle et al., 2021).

Contrary to our assumptions, beliefs in CAM did not predict OLP acceptance. CAM beliefs are primarily captured in the Holistic Health and Alternative Medicine Questionnaire through items emphasizing its differentiation from conventional medicine (e.g., “Complementary medicine should be subject to more scientific testing before it can be accepted by conventional doctors”; inverted item). The lack of predictive value for CAM beliefs may be explained by the fact that OLPs are difficult to categorize as either conventional medicine or CAM, since they are transparently administered and empirically supported, yet not part of standard medical practice.

Previous research has shown that the association between CAM and holistic health beliefs can be attributed to a higher-order factor related to holistic versus analytic processing (Hyland et al., 2003). Consistent with this finding, the present study revealed that participants who reported greater willingness to take OLPs scored lower on three thinking-style scales, including effortful thinking, actively open-minded thinking, and close-minded thinking. In other words, these individuals reported to be less inclined to invest cognitive effort in problem-solving, relied less on empirical evidence when forming attitudes, exhibited less black-and-white thinking, and demonstrated a stronger tendency to adhere to a single fixed explanation. This pattern aligns with the concept of holistic thinking, which is generally characterized by a focus on the big picture rather than on detailed analytical processing.

It is noteworthy that these findings reflect participants’ self-concept about their thinking styles but not necessarily their actual behavior. However, there is empirical evidence that placebo responders exhibit certain cognitive tendencies or biases. For example, participants who took placebos labeled as cognitive enhancers reported experiencing performance improvements (subjective perception of improvement) despite a lack of objective improvement (Schwarz & Büchel, 2015). This suggests a tendency to rely on emotional reasoning, motivated reasoning, and wishful thinking rather than empirical data, which may also be relevant in the context of OLP treatments (Trippas et al., 2015).

Finally, medical maximizing–minimizing and dispositional optimism did not emerge as unique predictors of OLP acceptance, although they did show statistically significant zero-order correlations. Medical maximizing–minimizing not only influences how frequently individuals seek medical interventions but is also associated with the quality of care received. Compared to minimizers, medical maximizers are more likely to pursue inappropriate medical procedures that are either ineffective or unnecessary (Mott et al., 2021; Scherer et al., 2020). However, our findings suggest that medical maximizing-minimizing is not linked to OLP acceptance. This aligns with previous survey results indicating that the tendency to avoid taking pills for health problems is unrelated to the willingness to take OLP pills (Schienle & Seibel, 2024).

Previous studies examining the impact of optimism on placebo effectiveness have yielded mixed results (Kern et al., 2020; Locher et al., 2019; Newton et al., 2024). While it seems reasonable to assume that optimism influences expectations of OLP effectiveness and consequently OLP acceptance, this concept may be too broad to fully account for attitudes and behaviors in healthcare settings.

Additional limitations of the present research need to be acknowledged. First, we investigated a convenience sample in Austria that predominantly consisted of young females with a high level of education. Therefore, the findings cannot be generalized to other sociodemographic groups (e.g., older or clinically relevant populations). Second, our findings are based on a regression analysis, which does not provide insight into causality. Third, our measure of holistic health beliefs demonstrated only borderline reliability. Fourth, we asked participants about their willingness to take an OLP and their thinking styles in an online survey. Actual OLP use as well as cognitive processing were not assessed. Finally, potential confounding variables, such as the presence of specific health conditions or negative experiences with the health care system were not assessed.

Building on the findings of the present study and considering its shortcomings, several avenues for future research emerge. First, prospective or experimental studies could examine causal relationships between thinking styles, health beliefs, and OLP acceptance, as the present cross-sectional design cannot establish directionality.

Second, research could explore whether interventions targeting health beliefs or analytical thinking might increase openness to OLP treatments. For example, psychoeducational programs could inform potential OLP users of everyday mental processes that produce physically measurable changes, such as the relationship between stress levels and antibody production. Furthermore, presentation of the scientific basis concerning placebo effects in a logically patient-centered manner might be able to reach skeptical-analytical thinkers (Evers et al., 2020).

Third, replication in more diverse populations—including different age groups, cultural/ethnic contexts, and clinical samples—would help determine the generalizability of these associations. For example, differences in laypeople’s attitudes toward medicine have been documented internationally and even between social milieus within individual countries (Horne et al., 2004; Ortiz et al., 2025).

Finally, investigating additional psychological and contextual factors, such as prior experiences with medication, or the healthcare system, could further elucidate the mechanisms underlying OLP acceptance and rejection.

## 5. Conclusions

This survey revealed that self-reported holistic thinking styles and holistic health beliefs were significantly associated with a greater reported willingness to take an OLP. In contrast, a more analytical thinking style and a stronger inclination toward conventional medicine were linked with OLP rejection. These associations should be interpreted with caution given the correlational and cross-sectional nature of the study. Future research should employ longitudinal and experimental-interventional designs to allow for causal inferences.

## Figures and Tables

**Table 1:** Internal consistencies, means, standard deviations, and Spearman’s correlation coefficients with open-label placebo acceptance, including 95% confidence intervals (*n* = 326).

Measure	Cronbach’s Alpha	Mean (SD)	Rho	95% CI Rho (LL; UL)	*p*
OLP acceptance	-	55.46 (32.98)			
MM1	-	3.21 (1.50)	−0.11 *	−0.22; 0.00	0.048
SOP-2 (optimism)	-	4.72 (1.43)	0.12 *	0.01; 0.23	0.024
Holistic Health and Alternative Medicine Questionnaire					
CAM	0.79	19.49 (6.10)	0.17 **	0.06; 0.27	0.002
Holistic health	0.68	25.47 (3.46)	0.23 ***	0.12; 0.33	<0.001
4-Component Thinking Style Questionnaire					
AOT	0.88	4.35 (0.98)	−0.23 ***	−0.33; −0.12	<0.001
CMT	0.82	2.39 (0.92)	−0.13 *	−0.24; −0.02	0.015
PET	0.82	4.68 (0.87)	−0.15 **	−0.25; −0.04	0.008
PIT	0.93	3.92 (0.94)	0.15 **	0.04; 0.26	0.006

Notes: A single asterisk (*), double asterisk (**), and triple asterisk (***) indicate statistical significance at *p* < 0.05, *p* < 0.01, and *p* < 0.001, respectively. “LL” and “UL” indicate the lower and upper limits of the 95% confidence intervals, respectively. Abbreviations: AOT: actively open-minded thinking; CAM: complementary and alternative medicine; CI: confidence interval; CMT: Closed-minded thinking; MM1: medical maximizer–minimizer elicitation question; OLP: open-label placebo; PET: preference for effortful thinking; PIT: preference for intuitive thinking; SD: standard deviation; SOP-2: Optimism–Pessimism Short Scale-2.

**Table 2:** Binary logistic regression for open-label placebo acceptance.

Step	Predictor	Estimate	SE	Exp(B)	Exp(B) 95% CI		*p*	R^2^
					Lower	Upper		
1	(Intercept)	0.38	0.15	1.46	1.12	2.07	0.014	0.03 ^a^
	Sex	0.61 ^a^	0.31 ^a^	1.85 ^a^	1.0 4 ^a^	3.69 ^a^	0.046 ^a^	
	Age	−0.02	0.01	0.98	0.96	1.00	0.050	
2	(Intercept)	0.50	0.17	1.65	1.24	2.46	0.004	0.18 ^a^
	Sex	0.48	0.35	1.61	0.90	3.52	0.170	
	Age	−0.02	0.01	0.98	0.96	1.01	0.213	
	MM1	−0.13	0.11	0.87	0.69	1.11	0.211	
	Optimism	0.16	0.11	1.18	0.94	1.52	0.145	
	Holistic Health and Alternative Medicine Questionnaire							
	CAM	0.04	0.03	1.04	0.97	1.11	0.154	
	Holistic health	0.14 ^a^	0.05 ^a^	1.15 ^a^	1.05 ^a^	1.30 ^a^	0.007 ^a^	
	The 4-Component Thinking Style Questionnaire							
	AOT	−0.42 ^a^	0.20 ^a^	0.65 ^a^	0.44 ^a^	0.97 ^a^	0.031 ^a^	-
	CMT	−0.47 ^a^	0.18 ^a^	0.63 ^a^	0.41 ^a^	0.92 ^a^	0.011 ^a^	
	PET	−0.47 ^a^	0.20 ^a^	0.62 ^a^	0.40 ^a^	0.91 ^a^	0.021 ^a^	
	PIT	−0.12	0.20	0.88	0.60	1.40	0.534	

Notes: In the analysis, open-label placebo acceptance scores ≤ 33 and ≥66 were recoded as 0 (rejection) and 1 (acceptance), respectively. ^a^ Significant predictor at *p* < 0.05. Sex was contrast-coded using simple coding (men = −0.5; women = 0.5); metric predictors are mean-centered; confidence intervals were computed using bootstrapping with bias-correction and acceleration based on 1000 repetitions. Abbreviations: AOT: actively open-minded thinking; CAM: complementary and alternative medicine; CI: confidence interval; CMT: closed-minded thinking; MM1: medical maximizer–minimizer elicitation question; PET: preference for effortful thinking; PIT: preference for intuitive thinking; SE: standard error.

## Data Availability

The raw data supporting the conclusions of this article will be made available by the authors on request.

## Supplementary Materials

The following supporting information can be downloaded at: https://www.mdpi.com/article/10.3390/bs16020198/s1, Table S1: The 4-Component Thinking Style Questionnaire; Table S2: Holistic Complementary and Alternative Medicine Questionnaire; Table S3: The Optimism–Pessimism Short Scale–2; Table S4: Maximizer-minimizer elicitation question.
