# Supplementary material for: Acceptance of Open-Label Placebos Is Linked to Holistic Health Beliefs and Thinking Styles
Source: Behav Sci (Basel). 2026 Jan 29;16(2):198. doi: 10.3390/bs16020198 (PMC12937914; doi:10.3390/bs16020198)
Supplement: Supplementary file 1 [file behavsci-16-00198-s001.zip › behavsci-3974359-supplementary.pdf]

Supplementary Material

Table S1: The 4-Component Thinking Style Questionnaire (4-CTSQ)

Subscale	Original item (Newton et al., 2024)	German version (own translation)
AOT	It is important to be loyal to your beliefs even when evidence is brought to bear against them. (R)	Es ist wichtig, seinen Überzeugungen treu zu bleiben, auch wenn Fakten dagegen sprechen. (R)
AOT	Whether something feels true is more important than evidence. (R)	Ob sich etwas wahr anfühlt, ist wichtiger als die Faktenlage. (R)
AOT	Just because evidence conflicts with my current beliefs does not mean my beliefs are wrong. (R)	Nur weil Tatsachen im Widerspruch zu meinen derzeitigen Überzeugungen stehen, bedeutet das nicht, dass meine Überzeugungen falsch sind. (R)
AOT	There may be evidence that goes against what you believe but that does not mean you have to change your beliefs. (R)	Es kann Fakten geben, die dem widersprechen, was man glaubt, aber das bedeutet nicht, dass man seine Überzeugungen ändern muss. (R)
AOT	Even if there is concrete evidence against what you believe to be true, it is OK to maintain cherished beliefs. (R)	Auch wenn es konkrete Belege gegen das gibt, was man glaubt, ist es in Ordnung, an liebgewonnenen Überzeugungen festzuhalten. (R)
AOT	Regardless of the topic, what you believe to be true is more important than evidence against your beliefs. (R)	Unabhängig vom Thema ist das was man für wahr hält wichtiger, als Beweise gegen die eigenen Überzeugungen. (R)
CMT	I think there are many wrong ways, but only one right way, to almost anything.	Ich glaube, es gibt viele falsche Wege, aber nur einen richtigen Weg zu fast allem.
CMT	In my experience, the truth is often black and white.	Meiner Erfahrung nach ist die Wahrheit oft schwarz und weiß.
CMT	Truth is never relative.	Die Wahrheit ist nie relativ.
CMT	The truth does not change.	Die Wahrheit ändert sich nicht.
CMT	Either something is true or it is false; there is nothing in-between.	Entweder ist etwas wahr oder es ist falsch, es gibt nichts dazwischen.
CMT	There is no middle ground between what is true and what is false.	Es gibt keinen Mittelweg zwischen dem, was wahr ist, und dem, was falsch ist.
PIT	I like to rely on my intuitive impressions.	Ich verlasse mich gerne auf meine intuitiven Eindrücke.
PIT	I believe in trusting my hunches.	Ich glaube, dass ich meinem Bauchgefühl vertrauen kann.
PIT	When I make decisions, I tend to rely on my intuition.	Wenn ich Entscheidungen treffe, neige ich dazu, mich auf meine Intuition zu verlassen.
PIT	Using my “gut-feelings” usually works well for me in figuring out problems in my life.	Mein Bauchgefühl hilft mir in der Regel gut, Probleme in meinem Leben zu lösen.
PIT	Intuition is the best guide in making decisions.	Intuition ist der beste Wegweiser bei der Entscheidungsfindung.

PIT	I often go by my instincts when deciding on a course of action.	Ich verlasse mich oft auf meinen Instinkt, wenn ich mich für eine bestimmte Vorgehensweise entscheide.
PET	I’m not that good at figuring out complicated problems. (R)	Ich bin nicht so gut darin, komplizierte Probleme zu lösen. (R)
PET	Thinking is not my idea of an enjoyable activity. (R)	Nachdenken ist für mich keine angenehme Aktivität. (R)
PET	I try to avoid situations that require thinking in depth about something. (R)	Ich versuche Situationen zu vermeiden, in denen ich intensiv über etwas nachdenken muss. (R)
PET	I am not a very analytical thinker. (R)	Ich bin kein sehr analytischer Denker. (R)
PET	Reasoning things out carefully is not one of my strong points. (R)	Es gehört nicht zu meinen Stärken, Dinge sorgfältig zu durchdenken. (R)
PET	Thinking hard and for a long time about something gives me little satisfaction. (R)	Lange und intensiv über etwas nachzudenken, gibt mir wenig Befriedigung. (R)

Note. R = reverse-scored items. Subscales: AOT = Actively Open-Minded Thinking; CMT = Close-Minded Thinking; PIT = Preference for Intuitive Thinking; PET = Preference for Effortful Thinking.

Response scales range from strongly disagree (1) to strongly agree (6).

Newton, C., Feeney, J., & Pennycook, G. (2024). On the disposition to think analytically: Four distinct intuitive-analytic thinking styles. *Personality and Social Psychology Bulletin*, *50*(6), 906-923. https://doi.org/10.1177/01461672231154886

Table S2: Holistic Complementary and Alternative Medicine Questionnaire (HCAMQ)

	Original item (Hyland et al., 2003)	German version (own translation)
HH	Positive thinking can help you fight off a minor illness	Positives Denken kann im Kampf gegen eine leichtere Krankheit helfen
CAM	Complementary medicine should be subject to more scientific testing before it can be accepted by conventional doctors (R)	Die Komplementärmedizin sollte stärker wissenschaftlich geprüft werden, bevor sie von Schulmedizinern akzeptiert werden kann. (R)
HH	When people are stressed it is important that they are careful about other aspects of their lifestyle as their body already has enough to cope with	Wenn Menschen gestresst sind, ist es wichtig, dass sie auf andere Aspekte ihres Lebensstils achten, da ihr Körper schon genug gefordert ist
CAM	Complementary medicine can be dangerous in that it may prevent people getting proper treatment (R)	Die Komplementärmedizin kann insofern gefährlich sein, da sie eine sachgerechte Behandlung verhindert. (R)
HH	The symptoms of an illness can be made worse by depression	Die Symptome einer Krankheit können durch eine Depression verschlimmert werden
CAM	Complementary medicine should only be used as a last resort when conventional medicine has nothing to offer (R)	Die Komplementärmedizin sollte nur als letztes Mittel eingesetzt werden, wenn das Wissen der Schulmedizin erschöpft ist (R)

HH	If a person experiences a series of stressful life events they are more likely to become ill	Wenn eine Person eine Reihe von belastenden Lebensereignissen erlebt, ist die Wahrscheinlichkeit größer, dass sie krank wird
CAM	It is worthwhile trying complementary medicine before going to the doctor	Es lohnt sich, Komplementärmedizin auszuprobieren, bevor man zum Arzt geht
CAM	Complementary medicine should only be used in minor ailments and not in the treatment of more serious illness (R)	Komplementärmedizin sollte nur bei leichten Beschwerden und nicht bei der Behandlung schwerwiegenderer Krankheiten eingesetzt werden (R)
HH	It is important to find a balance between work and relaxation in order to stay healthy	Es ist wichtig, ein Gleichgewicht zwischen Arbeit und Erholung zu finden, um gesund zu bleiben
CAM	Complementary medicine builds up the body's own defences, so leading to a permanent cure	Komplementärmedizin baut körpereigene Abwehrkräfte auf, und führt so zu einer langfristigen Widerstandsfähigkeit

Note. R = reverse-scored items. Subscales: HH: Holistic Health; CAM: Complementary and Alternative Medicine

Response scales range from strongly disagree (1) to strongly agree (6) and a sum score is computed for each of the subscales.

Hyland, M. E., Lewith, G. T., & Westoby, C. (2003). Developing a measure of attitudes: the holistic complementary and alternative medicine questionnaire. *Complementary therapies in medicine*, *11*(1), 33-38. https://doi.org/10.1016/s0965-2299(02)00113-9

Table S3: The Optimism–Pessimism Short Scale–2 (SOP2)

English Item version (Nießen et al., 2022)	German item version
The next question deals with optimism. Optimists are people who look to the future with confidence and who mostly expect good things to happen. How would you describe yourself? How optimistic are you in general?	Optimisten sind Menschen, die mit Zuversicht in die Zukunft blicken und meistens Gutes erwarten. Bitte schätzen Sie sich selbst ein: Wie optimistisch sind Sie im Allgemeinen?
The next question is about pessimism. Pessimists are people who are full of doubt when they look to the future and who mostly expect bad things to happen. How would you describe yourself? How pessimistic are you in general?	Pessimisten sind Menschen, die voller Zweifel in die Zukunft blicken und meistens Schlechtes erwarten. Bitte schätzen Sie sich selbst ein: Wie pessimistisch sind Sie im Allgemeinen?

Note. The pessimism score is reverse-scored to compute an optimism score using both subscales.

Responses were recorded on a 7-point scale ranging from 1 (not at all optimistic/pessimistic) to 7 (very optimistic/pessimistic).

Nießen, D., Groskurth, K., Kemper, C. J., Rammstedt, B., & Lechner, C. M. (2022). The Optimism–Pessimism Short Scale–2 (SOP2): A comprehensive validation of the English-language adaptation. *Measurement Instruments for the Social Sciences*, *4*(1), 1. https://doi.org/10.1186/s42409-021-00027-6

Table S4: Maximizer-minimizer elicitation question (MM1)

Original item (Scherer & Zikmund-Fisher, 2020)	German version (own translation)
**Thinking about how much healthcare you prefer to get: What type are you?**<br><br>Sometimes, medical action is clearly necessary, and sometimes it is clearly NOT necessary. Other times, reasonable people differ in their beliefs about whether medical action is needed.<br><br>In situations where it is not clear, do you tend to lean towards **taking action** or do you lean towards **waiting and seeing** if action is needed?<br><br>**Importantly, there is no "right" way to be.**	**Wenn Sie darüber nachdenken, wie viel medizinische Versorgung Sie wünschen: Welcher Typ sind Sie?**<br><br>Manchmal sind medizinische Maßnahmen (z.B. Tabletteneinnahme, Arzttermine) eindeutig notwendig und manchmal sind sie eindeutig nicht notwendig. Menschen unterscheiden sich in ihrer Meinung, ob medizinische Maßnahmen erforderlich sind. Neigen Sie in Situationen, in denen dies nicht klar ist dazu, **Maßnahmen zu ergreifen** oder **warten Sie lieber ab**?<br><br>**Wichtig ist, dass es keine „richtige" Art und Weise gibt.**

The response scale ranges from 1: I strongly lean toward waiting and seeing to 6: I strongly lean toward taking action (Ich neige stark dazu abzuwarten/ ich neige stark dazu, zu handeln)

Scherer LD, Zikmund-Fisher BJ. Eliciting Medical Maximizing-Minimizing Preferences with a Single Question: Development and Validation of the MM1. *Medical Decision Making*. 2020;40(4):545-550. doi:10.1177/0272989X20927700
